# Limited Plasticity of Prismatic Visuomotor Adaptation

**DOI:** 10.1177/2041669517701458

**Published:** 2017-04-10

**Authors:** Karoline Spang, Sven Wischhusen, Manfred Fahle

**Affiliations:** Department of Human-Neurobiology, University of Bremen, Germany

**Keywords:** body perception, multisensory or cross-modal processing, perceptual learning, pointing or hitting, prism adaptation, visuohaptic interactions

## Abstract

Movements toward an object displaced optically through prisms adapt quickly, a striking example for the plasticity of neuronal visuomotor programs. We investigated the degree and time course of this system’s plasticity. Participants performed goal-directed throwing or pointing movements with terminal feedback before, during, and after wearing prism goggles shifting the visual world laterally either to the right or to the left. Prism adaptation was incomplete even after 240 throwing movements, still deviating significantly laterally by on average of 0.8° (CI = 0.20°) at the end of the adaptation period. The remaining lateral deviation was significant for pointing movements only with left shifting prisms. In both tasks, removal of the prisms led to an aftereffect which disappeared in the course of further training. This incomplete prism adaptation may be caused by movement variability combined with an adaptive neuronal control system exhibiting a finite capacity for evaluating movement errors.

## Introduction

Prism adaptation serves as an experimental paradigm to study the mechanisms of senso-motor plasticity that exists even in the adult brain, as do priming and perceptual and sensorimotor learning (Ahissar & Hochstein, 2004; Fahle & Poggio, 2002; Gilbert, Sigman, & Crist, 2001).

### Phenomenology of Prism Adaptation

Prisms shift the visual world, causing a mismatch between perceived object position and arm movement trajectory. Subjects wearing prism goggles perform movements toward a visual target (e.g., pointing, reaching, or throwing) that are initially offset due to the prism shift (Martin, Greger, Norris, & Thach, 2001; Martin, Keating, Goodkin, Bastian, & Thach, 1996a; Martin, Norris, Greger, & Thach, 2002; Norris, Greger, Martin, & Thach, 2001; Redding & Wallace, 2003b; von Helmholtz, 1867). Within a few trials under visual feedback, the error decreases substantially since subjects adapt to the optical displacement possibly due to both *cognitive* and *automatic* mechanisms (Bedford, 1999; Fernandez-Ruiz & Diaz, 1999; Harris, 1963, 1965, 1980; Held & Freedman, 1963; Redding, Rossetti, & Wallace, 2005; Redding & Wallace, 2003a).

After removal of the prisms, movements initially deviate in the direction opposite to the prismatic displacement, indicating a negative aftereffect probably based on an automatic mechanism and which vanishes in the course of further visuomotor practice (Harris, 1965, 1980; Newport, Brown, Husain, Mort, & Jackson, 2006; Newport & Jackson, 2006; Redding et al., 2005; Redding & Wallace, 2002).

### Mechanism and Neural Substrates of Prism Adaptation

Both the exact mechanisms and underlying neural substrates of prism adaptation remain unclear (Block & Bastian, 2012; Shadmehr & Wise, 2005). The long-held view that prism adaptation is based purely on adaptation of hand proprioception (Harris, 1963) must be revised in the light of recent evidence showing no transfer of adaption between pointing and throwing (Wischhusen, 2008) and between different fingers of the same hand (Schot, Brenner, & Smeets, 2014).

### Speed and Extent of Prism Adaptation

In most studies examining prism adaptation in humans, the number of trials during the adaptation period varied between 20 and 40 movements (Fernandez-Ruiz, Hall, Vergara, & Diiaz, 2000; Fernandez-Ruiz et al., 2003; Fernandez-Ruiz et al., 2000; Kitazawa, Kohno, & Uka, 1995; Martin et al., 2001; Martin, Keating, Goodkin, Bastian, & Thach, 1996b; Roller, Cohen, Kimball, & Bloomberg, 2001). Typically, this period of visuomotor training was sufficient for adaptation to take place since error reduction occurred and aftereffects were observed upon removal of the prisms. Since aftereffects are considered as the measure of *true* adaptation, the attention of previous studies was not directed to the level of adaptation in the prism condition itself after training. In pilot experiments, however, we noticed that adaptation for throwing seemed to be incomplete. We wondered whether this incompleteness of adaptation was due to an insufficient number of trials during the adaptation period and therefore substantially increased the number of trials to ensure sufficient visuomotor training. To control for effects of task specificity, we employed two different visuomotor tasks, namely pointing and throwing. These two tasks test movements of different skills and speed at different distances and with a differing role of proprioception. Our results demonstrate incomplete adaptation for throwing even after extensive practice and less so for pointing. A similar incompleteness of adaptation during throwing movements appears in graphs of previous studies (Fernandez-Ruiz & Diaz, 1999; Martin et al., 1996a) but was not addressed by these authors. To the best of our knowledge, this incompleteness of adaptation with prisms has so far not received any attempt at explanation. We here suggest a mechanism for incomplete prism adaptation based on Wischhusen’s doctoral thesis (2008) and very much in line with a recent suggestion by van der Kooij, Brenner, van Beers, and Smeets (2015) based on adaptation to changes of visual feedback to a hand hold cube and with state space models (Cheng & Sabes, 2006; Smith, Gazizadeh, & Shadmehr, 2006).

## Materials and Methods

### Ethics Statement

Subjects were informed about the general aim and procedure of the experiment but not its exact purpose and had to sign a written consent form. The tenets of the Declaration of Helsinki were strictly observed. Some subjects were students of the University and were unpaid; the others were paid for participation in the experiment. All were free to withdraw from the study at any time. The study was approved by the ethics committee at the University of Bremen and done in full compliance with the guidelines of this committee.

### Experimental Setup and Procedure

**Figure 1.**
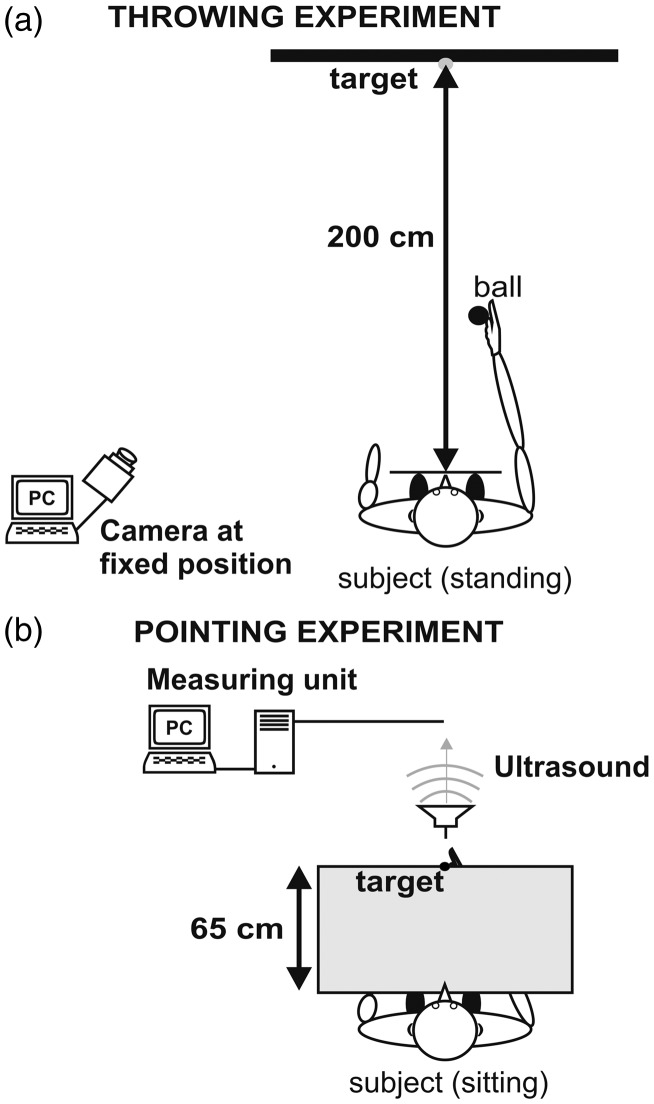
Experimental setup. (a) Throwing Experiment, in which subjects stood upright in front of a Velcro layered wall at a distance of 2 m. Subjects’ body midline was centered with the target. The task was to throw a ball to the target. After each throw, a photo was taken using a digital camera. (b) In the Pointing Experiment, subjects were sitting at a table and performed pointing movements underneath an opaque table toward a visual target which was fixed at the table’s edge. Subjects only had terminal visual feedback about their index fingers position at the end of the trajectory. A PC-driven ultrasound measuring unit was placed in front of the table for assessing the movements’ spatial accuracy. Target and subject’s index finger were equipped with small ultrasound transmitters.

In the *Throwing Experiment*, subjects had to throw softballs (24 g, 5.0 cm diameter) toward a central visual target on a 1.5 m × 1.5 m wide wall. The target was a blue spot (2.0 cm diameter) attached to the wall at a height of 156 cm. Subjects stood upright with their mid-sagittal plane aligned with the target at a viewing distance of 2.0 m (Figure 1(a)). With their head being unrestrained, subjects saw the target under daylight illumination. The spatial offset between the target and the impact of the ball was clearly visible after each throw, providing immediate visual feedback about the precision of the throw. The wall was layered with Velcro material, and the softball adhered to the wall after each throw. The wedge prisms of high optical quality (Carl Zeiss, Oberkochen, Germany) shifted the visual image laterally by ∼16° either to the right or left, and subjects immediately experienced a shift in their visual world when putting on the prism goggles. Subjects had to perform overhand throws using the right arm. After a brief instruction, subjects performed about 10 test throws to get used to the task without prisms.

The Throwing Experiment consisted of three conditions in the following order: pre-prism condition (PRE; including the baseline measurement, with prisms off), prism condition (PRISM; adaptation, with prisms on), and post-prism condition (POST; aftereffect, with prisms off). Depending on the protocol in the various experimental groups (Table 1) either 60 throws or else 120 throws were performed in the PRE condition. In the PRISM condition, testing the adaptation process, either 120 throws or 240 throws served to ensure extensive training. The POST condition consisted of either 90 or else 120 throws. Between conditions, subjects kept their eyes closed.

**Table 1.** Detailed Protocol and Number, Age, and Gender of Subjects Participating in the Subdivisions of the Throwing (T) and Pointing (P) Experiments.

Task	Group	Investigator	Prism	Number of events			Subjects		
				PRE^a^	PRISM^a^	POST^a^	*n*	Age^b^	Gender Female/Male
Throwing	T1	1	Right 17°	120	120	120	8	23.5 ± 2	7/1
	T2	1	Left 17°	120	120	120	8	23.4 ± 1	5/3
	T3	1	Right 17°	60	240	90	10	24.5 ± 2	5/5
	T4	2	Right 17°	60	240	90	6	24.5 ± 4	6/0
	T5	2	Left 17°	60	240	90	6	24.3 ± 2	5/1
Pointing	P1	1	Right 17°	120	120	120	10	23.9 ± 2	7/3
	P2	1	Left 17°	120	120	120	10	24.1 ± 2	9/1

*Note.* ^a^PRE = without prisms, PRISM = prisms on, POST = prisms off.

b Age: mean and *SD*.

In the *Pointing Experiment*, subjects performed fast pointing movements toward a visual target using the right arm underneath the (opaque) table (Figure 1(b)). Subjects were sitting at the table with their mid-sagittal plane aligned with the table, looking at a target which was a small white disk fixed at the table’s edge at a distance of 65 cm from the subject. The table-top was opaque; hence, subjects could not see and hence not correct their movements on-line during the movement based on visual feedback during the trajectory. The trajectory of the arm therefore was purely feed-forward regarding visuohaptic mismatch. Subjects viewed the target binocularly and had terminal visual feedback about the position of the index finger only at the end of the arm movement. The movements’ trajectory was assessed using a PC-driven ultrasound measuring system operating with high spatial resolution (Zebris medical, Isny, Germany). Ultrasound signals were sent out by small transmitters attached to the index finger of the right pointing hand and by the target (measuring rate: 100 Hz, dimensions *x-y-z*). Similar to the Throwing Experiment, the Pointing Experiment consisted of a PRE, PRISM, and POST condition, each with 120 movements (Table 1). Subjects were instructed to fixate the target and to keep the eyes closed between conditions. The wedge prisms were the same as in the Pointing Experiment.

### Experimental Groups and Subjects

Forty-four females and fourteen males (mean age: 24 years) were subdivided into seven experimental groups of right-handed humans. The exact experimental protocols are indicated in Table 1. Subjects reported no history of brain or eye disorders and had normal or corrected-to-normal visual acuity.

### Data Analysis

All distances mentioned in this manuscript are horizontal distances between the central target and the endpoint of the movement in degrees of visual angle (Throwing Experiment: position of the ball on the wall; Pointing Experiment: top of index finger at the end of the trajectory).

For the Throwing Experiment, data were collected with a digital camera and analyzed with software developed in-house based on Matlab. In the Pointing Experiment, a posthoc analysis yielded the horizontal distance between movement endpoints and the target from the Zebris recordings excluding any correction movements. For further analysis, all data were baseline corrected for each subject individually. That is, we subtracted the mean of deviations in the last 30 pointing or throwing movements in the PRE condition (the *BASELINE*) from *each* data point (Clower & Boussaoud, 2000). Baseline correction normalized the data by removing any spatial bias, for example, caused by parallax influence of the dominant eye. To analyze the data in terms of completeness of the adaptation process, we divided each condition (PRE, PRISM, and POST) into blocks of 30 subsequent throws. Blocks were averaged across subjects and tested against the value of zero (i.e., no deviation from normalized target) using a one-sided *t* test since we expected incomplete adaptation. In the analysis, we focused on the last block of the PRISM and POST conditions since this value reflects the *remaining* lateral deviation. Specifically, a last block of the PRISM condition differing significantly from zero indicates a remaining lateral deviation at the end of the adaptation period, hence suggesting incomplete adaptation.

In addition, we fitted an exponential function (see Appendix) as well as a double exponential function *f*(*x*) = *a* × *b*^(*c*^*x*) to the averaged time courses of adaptation (PRISM condition) and readaptation (POST condition) for the groups. The term *a* in this function represents the offset the function is approaching after an infinite number of throws, the parameters *b* and *c* represent the form and magnitude of the decay. The variable *x* denotes the trial number.

## Results

### Throwing

#### Time courses and double exponential fits

Throws in the PRE condition, including the baseline, were rather precise. The overall mean deviation for throws of all subjects in all experiments amounted to −0.16°, and the square root of mean of the variances was 2.2° (Baseline data can be found in Appendix Table A1.)

**Figure 2.**
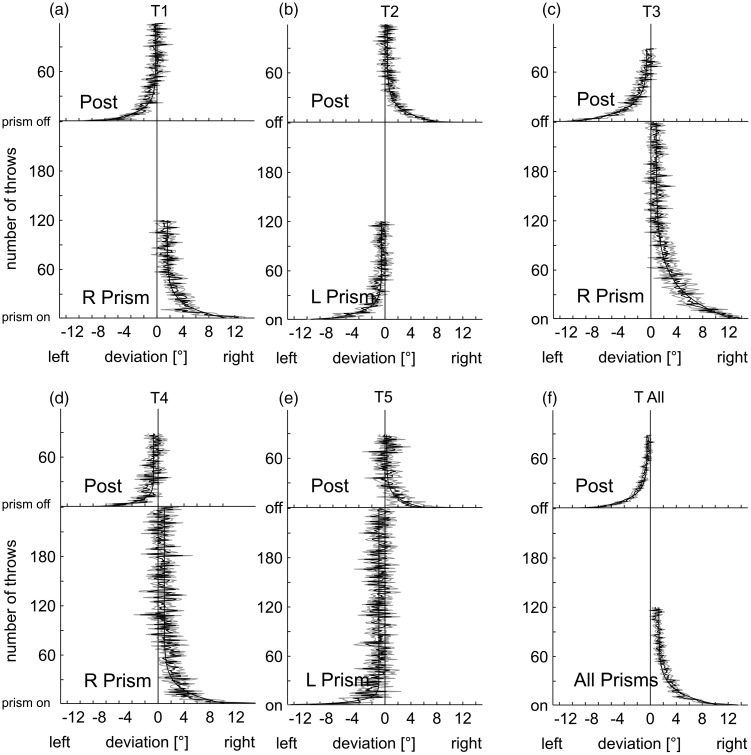
(a) to (f) Lateral deviations of movements’ endpoints from the target in degrees of visual angle in the PRISM and POST conditions as a function of movement number. (a) to (e) Throwing Experiments T1 to T5. (f) Grand average T_all_. Note: Positive values on the abscissa indicate a rightward deviation from the target whereas negative values denote a leftward one, except for T_all_, where data for the leftward shifting prisms were multiplied by −1. Averaged results T all include only blocks that were performed by all groups. All data shown are baseline corrected (means and *SEM*).

Averaged baseline-corrected time courses of prism adaptation and readaptation of all experimental groups T1 to T5 are presented in Figure 2(a) to (e). In addition, we display a grand average for all experimental groups (T_all_; Figure 2(f)).

At the beginning of the PRISM condition, movements deviated corresponding to the direction of the prismatic deflection introduced (see Appendix Table A2 for size of initial prism effects of all groups). The initial deviation of the first throw was on average 14.0° or 87.5% of the optical deviation of the rightward shifting prisms (*n* = 24) and −12.7° or 80% for the leftward shifting prisms (*n* = 16). The initial negative aftereffect of the first throw in the POST condition was −11.0° or 69% (right) and 10° or 63% (left) of the prism deviation of ∼16°. Hence each experimental group showed a marked lateral deviation of movements in the direction of the prismatic displacement when the prisms were introduced and an aftereffect in the opposite direction when the prisms were removed.

The average of the individual double exponential fits to the PRISM condition for each subject shows a constant offset (for number of throws converging to infinity) that is positive for right shifting prisms and negative for left shifting prisms, on average 0.8° (CI = 0.20°). Exact values including confidence intervals at the 95% level are listed in Table 2 (for fits of simple exponential functions of group means, see Appendix Table A3).

**Table 2.** Averages and Grand Average of the Double Exponential Least Square Fits of All Subjects.

Task	Group	*N*	Prism	PRISM condition		POST condition	
				*a* ^a^	CI^b^	*a* ^a^	CI^b^
Throwing	T1	8	Right	1.49	0.34	−0.18	0.10
	T2	8	Left	−0.56	0.37	0.23	0.11
	T3	10	Right	0.39	0.26	−0.28	0.17
	T4	6	Right	0.95	0.36	−0.62	0.29
	T5	6	Left	−0.77	0.75	0.44	0.35
	T_all_	38		0.80	0.20	0.33	0.09
Pointing	P1	10	Right	0.0006	0.48	−0.56	0.63
	P2	10	Left	−0.44	0.34	0.25	0.28
	P_all_	20		0.22	0.30	0.40	0.34

*Note.* The fitted equation is *f*(*x*) = *a* × *b*^(*c*^*x*); *b* is approx. 10 for PRISM (30 for POST); *c* is approx. 0.9 for both, hence *f*(*x*) decreases with increasing *x*.

a The parameter *a* corresponds to the remaining offset after an infinite number of throws or pointing movements, respectively.

b One-sided CI = confidence interval at the 95% confidence level over fits to all individual observers.

#### Completeness of prism adaptation: block analysis

**Figure 3.**
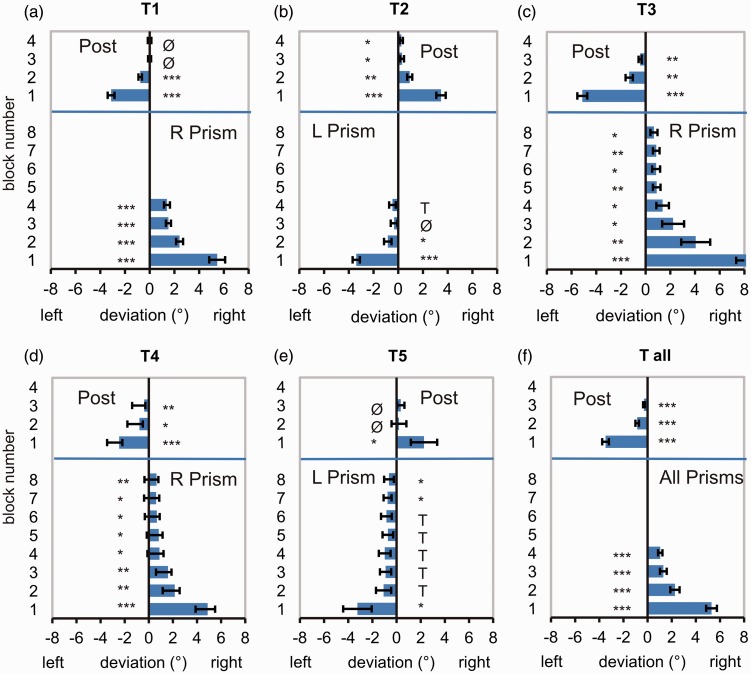
Block analysis of movements’ mean lateral deviation from the target in the PRISM and POST conditions. (a) to (e) Throwing Experiments T1 to T5. (f) Grand average T_all_. For each subject, throws were merged into blocks of 30 subsequent movements, and block means were calculated. Data shown represent group averages (means and *SEM*). The level of significance is marked by the number of asterisks. Asterisks indicate blocks differing significantly from zero as revealed by a one-sided *t* test (uncorrected).

Block-averaged baseline-corrected data of all experimental groups are presented in Figure 3(a) to (f). Individual mean deviations of all blocks and all experiments with the corresponding *p* values are listed in Appendix Table A4. For example, Block 1 (Throws 1–30) in the PRISM as well as in the POST condition differs significantly from zero (*p* < .05) in each of the five experimental groups and in the overall average (T_all_). Adaptation was fast initially, slowing down thereafter but even the last blocks of the PRISM condition show a significant or highly significant remaining deviation, with one exception (T2) that only shows a trend. The mean remaining error is 1.05° (± 0.18° *SEM*) after 120 throws and around 0.6° after 240 throws (see Table 3). Readaptation for both prisms is relatively slow in some subexperiments resulting in a remaining significant mean error opposite to the direction of the prism shift in Block 3 (Throws 61–90) in three of the five subexperiments. In the grand average T _all_, the mean of Block 3 is much smaller than in the PRISM condition but differs still highly significantly from zero (−0.3° ± 0.1° *SEM*; *p* = .00013).

**Table 3.** Group Means and *SEM* Deviations in Degrees of the Last Block in the PRISM Condition of the Throwing and Pointing Experiments. *T* and *P* Values Are the Result of a One-Sided *t* Test (No Bonferroni Correction). All Data Are Baseline Corrected.

Group	Last block^a^	Prism^b^	*N*	Mean	*SEM*	*t*	*p* (one sided)	Significance
T1	B4	Right	8	1.40	0.24	5.82	.0003	***
T2	B4	Left	8	−0.47	0.27	−1.74	.0631	T
T3	B8	Right	10	0.67	0.29	2.35	.0215	*
T4	B8	Right	6	0.64	0.16	4.06	.0049	**
T5	B8	Left	6	−0.63	0.40	−1.56	.0902	T
T_all_	B4	Right+Left	38	1.05	0.18	5.79	.0000	***
P1	B4	Right	10	−0.19	0.46	−0.42	.3432	Ø
P2	B4	Left	10	−0.18	0.29	−0.61	.2791	Ø
P_all_	B4	Right+Left	20	−0.01	0.27	−0.03	.4881	Ø

*Note.* T = trend < .1.

a Blocks include 30 subsequent movements (B4 = movements 91–120; B8 = movements 211–240).

b Prism = Direction of prismatic shift.

* *p* < .05. **p < .01. ***p < .001.

In summary, all groups of the Throwing Experiment show a decrease of mean lateral deviation in the PRISM condition over time but with a significant remaining error suggesting that subjects do not regain baseline-like motor accuracy in the course of training.

### Pointing

#### Time courses and double exponential fits

In the PRE condition, the mean lateral deviation for pointing movements of all subjects (*n* = 20) in both experiments amounted to −0.68°. The square root of mean of the variances was 1.7°, that is, somewhat lower than in throwing (2.2°). The mean of the last 30 pointing movements and standard deviations of each individual subject in the PRE conditions did not differ much from the overall mean. The mean individual deviations from the central target (0°) were generally measured as being higher in pointing than in throwing, possibly an artifact due to parallax and associated with the fact that targeting uses one eye rather than a cyclopean retina. This fact plays a lesser role at the 200 cm distance used in throwing than at 65 cm pointing distance. Possible artifacts and biases were compensated for individually by the baseline correction we used (see Appendix Table A1).

**Figure 4.**
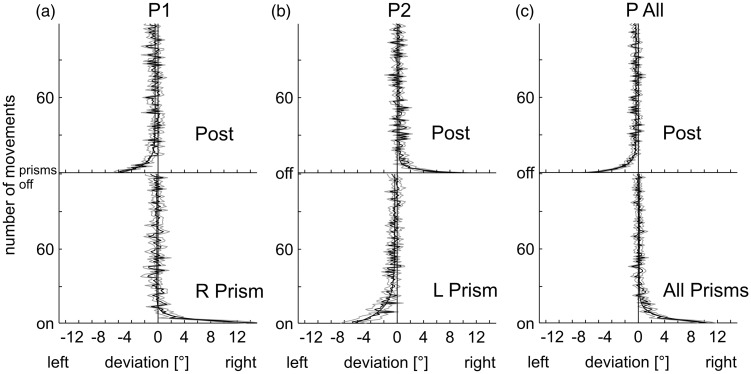
Lateral deviations of movements from the target in degrees of visual angle in the PRISM and POST conditions as a function of movement number in the Pointing Experiments. (a) Experiment P1 with a prism shift to the right, (b) Experiment P2 (prism shift left), and (c) Grand average P_all_ (*n* = 20). Positive values on the abscissa indicate a rightward deviation from the target whereas negative values denote a leftward one. All data shown are baseline corrected (means and *SEM*).

Averaged and baseline-corrected time courses of prism adaptation and readaptation of both groups P1 and P2 and the grand average (P_all_) are presented in Figure 4(a) to (c). At the beginning of the PRISM condition, movements deviated corresponding to the direction of the prismatic deflection and were larger for rightward (14.4° ± 1.1 *SEM*) than for leftward (−6.6° ± 2.0 *SEM*) shifting prisms (Appendix Table A2). On average (*n* = 20), the effect constituted 10.5° or 66% of the optical deviation of the prisms. The initial negative aftereffect of the first pointing movement averaged −7.8° or 49% of the optical prism deviation of 16°. Hence the initial errors of the PRISM and POST conditions are in the expected direction but smaller than in the Throwing Experiment. The individual double exponential fits to the adaptation curves produce confidence intervals including zero (Table 2); hence, the remaining error is not significant for the combined data but at least points in the expected directions and is significant for the left shifting prisms.

#### Completeness of prism adaptation: block analysis

**Figure 5.**
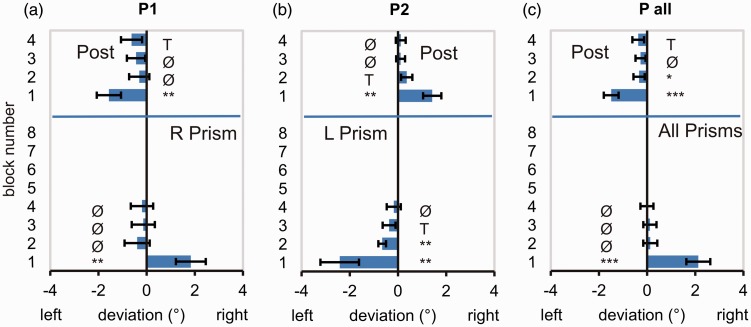
Block analysis of movements’ mean lateral deviation from the target in the PRISM and POST conditions in the Pointing Experiments. (a) Experiment P1 (prism shift right), (b) Experiment P2 (prism shift left), and (c) Grand average P_all_ (*n* = 20). For each subject, pointing movements were merged into blocks of 30 subsequent movements, and block means were calculated. Data shown represent group averages (means and *SEM*). Asterisks indicate blocks differing significantly from zero as revealed by a one-sided *t* test (uncorrected).

In the PRISM condition, both groups of the Pointing Experiment with right or left shifting prisms show a similar pattern of results (Figure 5(a) to (c)). While the first blocks of 30 movements all exhibit a considerable lateral deviation due to the prismatic deflection, the lateral deviation does not reach significance for the third and the fourth block. (The lack of significance may be due to the fact that block analysis is less sensitive than the fitting procedure described earlier.) Group mean deviations of last blocks of all experiments and the corresponding *p* values are listed in Table 3 (see Appendix Table A4 for all blocks).

## Discussion

We examined the effects of extensive training of a visuomotor task on the completeness of prism adaptation in human subjects and found that adaptation was incomplete, deviating significantly from the target in the Throwing Experiment, for both right- and leftward shifting prisms. Adaptation in the Pointing Experiment was more complete, with an error remaining at the end of the adaptation process that was significant only for leftward pointing prisms (see Table 2).

### Basics: Direct Effect, Prism Adaptation, and Aftereffect

Accurate preexposure visuomotor performance was evident in all groups with an average lateral deviation of −0.16° and a square root of mean of the variances of 2.2° for throwing. Corresponding values for pointing were −0.68° and 1.7°. Individual baseline correction is especially important for pointing due to possible influence of parallax. Contrary to our expectation, variance was only slightly larger for throwing than for pointing.

Introducing prisms initially shifted movements away from the target, *the direct effect*, but subjects rapidly recovered visuomotor accuracy in the course of training. During exposure, the movements’ lateral deviation decayed following a double exponential function. A simple exponential function delivered similar results (Appendix Table A3) but a lower fit.

This process of *adaptation* is supposed to rely on a fast strategic recalibration mechanism with postural adjustments and a slower realignment mechanism (Redding & Wallace, 2002). In all groups, the reduction of the lateral deviation in the PRISM condition was pronounced and statistically reliable (see Figure 2 for Throwing; Figure 4 for Pointing). As expected and well documented by earlier studies, a distinct *negative aftereffect* in the direction opposite to the prismatic displacement occurred when the prisms were removed.

### Prism Adaptation Can Be Incomplete

Precise preexposure visuomotor performance, a pronounced prism effect, and a significant error reduction during exposure as well the appearance of a negative aftereffect are well-documented results in the prism adaptation literature—so what is the main novel topic of our study?

The point is that even extensive training did *not* yield complete adaptation, with a significant remaining error especially for *throwing* movements. Although subjects were able to reduce their movements’ lateral error in the course of the PRISM condition, accuracy did not reach preexposure levels even after extensive throwing training. Movements deviated systematically in the direction of the prismatic displacement throughout the adaptation period—irrespective of whether this period covered 120 or even 240 throws. The asymptotic adaptation curves differ from zero (Figure 2) *and* the mean deviations of Throws 91 to 120 are in the direction of the prismatic shift in 33 out of the 38 subjects participating in the Throwing Experiments.

The double exponential decay function fitted to the averaged data (see Table 2) also demonstrates that the results of the PRISM condition asymptotically approach not zero deviation but maintain a certain remaining error, that is, they did not reach pretraining accuracy. The offset of the fitted decay function for the grand average (T_all_) amounted to 0.8°—with the 95% CI stretching to 0.6°. At first glance, this does not seem to be a large effect but note that 0.8° is the value the function is approaching to for an infinite number of throws. An offset of 0.8° corresponds to 2.8 cm at a distance of 200 cm—an offset which is clearly visible and should be corrected.

In contrast, the same mathematical procedure applied to the data of the *pointing* experiments yielded an offset of only 0.2° (with the 95% CI including zero) for the grand average P_all_, equivalent to a deviation of 0.17 cm. Still, the pointing deviations were in the expected directions, and the remaining errors were significant for the left shifting prisms.

### Possible Reasons for Differences Between Pointing and Throwing

Possible explanations for the task-related difference in the completeness of prism adaptation include different mechanisms of adaptation for near versus far space, for slow versus fast movements and the fact that throwing involves an object, while pointing does not (Berberovic & Mattingley, 2003; Previc, 1998). Moreover, there may be a less direct coupling between visual feedback regarding external objects (the position of the ball on the wall) versus body parts (position of the hand), and visual feedback is delayed due to the ball’s flight time (Kitazawa et al., 1995). Finally, the differences may mirror the differences between a high-level skill (pointing) and a low-level one (throwing).

### Basic Mechanisms of Adaptation Control

Several basic factors influence whether a subject comes to the *conclusion* that (s)he has sufficiently adapted their sensory motor representation or not. The first is the *variance* of motor performance. A high variance of movement or throwing endpoints will tend to mask a remaining systemic deviation in one direction. The second factor is the *memory* capacity and the ability to average over subsequent movement outcomes. The longer this memory and the more data are taken into account, the better will be the detection of a remaining deviation. The third factor is the level of *confidence* or the error probability chosen by the subject: A lower level of confidence for the detection of a mean deviation will lead to a smaller remaining error. (The subject will accept a *trend* toward one direction as requiring further adaptation.) In addition, considerations of the cost or reward ratio may play a role. Unfortunately, we can only directly measure the first of these factors, the variance of movement endpoints.

### A Hypothetical Model

Throwing variability limits complete prism adaptation if the neuronal integration capacity for target positions in extrapersonal space is finite. In general, even skilled or well-practiced movements vary from trial-to-trial, yielding a certain degree of movement variability which may result from variability during the execution of a specific movement (van Beers, Haggard, & Wolpert, 2004) or from variability during motor preparation or from both (Churchland, Afshar, & Shenoy, 2006).

We hypothesize that the size of the remaining error depends upon a neural error signal that subjects generate by integrating throwing deviations from target over a number of sequential trials (Wischhusen, 2008). However, this variability as such would only slow down adaptation but cannot by itself explain the large residual error. It is important in this context that each individual’s (visual) system has a finite *integration capacity* (see also Patzelt, Riegel, Ernst, & Pawelzik, 2007; Arevalo et al., 2013) to integrate deviations from target within a temporal interval in order to form an error signal. If we adopt the reasonable assumption that the system has a limited integration capacity over a number of throws (*n*), given a chosen error probability (α), the variance of throws (δ^2^) determines the point in time at which adaptation stops since the system is no longer *sure* that additional adaptation is required. This is the case if the confidence interval calculated on the basis of δ^2^ and *n* includes zero deviation. On the basis of a given Type I error (α = 0.05, CI = 1.95 δ), *n* can be calculated by the formula *n* = (1.95 δ/*a*) ^2 where *a* is the remaining offset of the PRISM condition, and δ is its standard deviation. Given the values of *a* = 0.8° and δ = 0.76° for throwing (Table 2), the system would detect a remaining deviation only if its integration capacity is at least *n* = 4. Integration capacity for throwing may be limited by the variation of throwing endpoints not only in horizontal but also in vertical direction. The corresponding values for pointing are *a* = 0.22° and δ = 0.82°, indicating an *n* of around 50 or else a less conservative error probability. For α = 0.2, the corresponding *n* = (1.3 δ/*a*) ^2 ≈ 25 movements. A better comparison between integration capacities may be absolute time rather than number of movements, given the eightfold longer intervals between throwing movements. Then, integration capacity is around 30 seconds for throwing and 50 seconds for pointing.

This model fits well with experiments on eye-hand-coordination in balancing a (virtual) stick. Patzelt et al. (2007) modeled human control dynamics for this system by assuming that the memory of humans for on-line adaptation is limited. Our models also fit well with the finding and arguments by van der Kooij et al. (2015) who investigated adaptation to spatial differences between a handheld object and its virtual representation. Moreover, we found an old article by Efstathiou (1969) who reports that there are no after-effects (and hence there is no prism adaptation according to the generally accepted definition of prism adaptation) for prisms displacing the visual image by up to 3° of visual angle. We interpret this as an additional argument that *small* average deviations from the intended target are not reliably detected and hence do not lead to (further) adaptation of arm or hand movements.

In addition to incomplete adaptation, we find that prism adaptation leads to a small but significant lasting disturbance of visuomotor control even after putting down the prisms. While the remaining error after readaptation is much smaller than the remaining error during adaptation—reflecting the fact that *proven* senso-motor functions are more stable than newly acquired ones, the remaining error at the end of the readaptation phase is still significant in several groups of subjects, indicating a longer than a short-lived distribution of the visuomotor control.

## Conclusion

In the present study, we demonstrate that prism adaptation is incomplete at least under some conditions. The incompleteness of adaptation with a remaining error that is larger for throwing than for pointing movements may be attributed to visuomotor variability coupled with a limited capacity to integrate errors over time and thus to evaluate the remaining mean error. Failure to measure accurately the remaining error obviously limits the extent of compensation through training. This phenomenon may also explain incomplete adaptation in other types of sensorimotor experiments.

## Supplementary Material

Supplementary material

## Appendix

**Table A1.** Individual Means (+*SD*) and Group Means (+*SEM*) of the Deviations of All Observers in the PRE Condition Used for Baseline Correction of the *Throwing* and *Pointing* Experiments.

Pre condition					
Group^a^	Subject	Baseline mean^b^	Baseline *SD*	Group BL mean	Group BL *SEM*
T1	1	−0.60	1.95		
	2	0.24	2.23		
	3	−0.40	1.95		
	4	−0.14	1.90		
	5	−0.19	2.12		
	6	−1.24	2.48		
	7	−0.12	1.89		
	8	−0.10	1.40		
T1_all_				−0.32	0.17
T2	1	0.00	2.12		
	2	−0.25	1.30		
	3	−0.26	1.02		
	4	0.12	2.67		
	5	−0.32	1.57		
	6	−1.20	1.36		
	7	−0.38	1.20		
	8	0.53	1.72		
T2_all_				−0.22	0.19
T3	1	−0.61	1.71		
	2	0.16	1.87		
	3	−0.14	1.75		
	4	0.23	1.69		
	5	−0.03	4.90		
	6	0.17	1.55		
	7	0.07	1.54		
	8	0.02	1.19		
	9	−0.34	1.64		
	10	−0.28	1.34		
T3_all_				−0.08	0.09
T4	1	−0.22	2.33		
	2	0.75	2.42		
	3	−0.65	3.34		
	4	0.10	2.17		
	5	−0.68	1.71		
	6	−0.38	2.15		
T4_all_				−0.18	0.24
T5	1	−0.62	2.75		
	2	−0.24	2.62		
	3	0.86	2.25		
	4	0.01	2.26		
	5	−0.43	2.38		
	6	0.39	3.61		
T5_all_				−0.01	0.25
P1	1	2.63	2.07		
	2	−2.00	1.14		
	2	0.75	1.49		
	4	0.34	1.72		
	5	−3.56	1.83		
	6	−2.58	1.76		
	7	1.14	1.21		
	8	−2.79	1.83		
	9	−2.86	1.52		
	10	−2.17	1.31		
P1_all_				−1.11	0.71
P2	1	−0.65	1.85		
	2	−0.22	1.64		
	2	0.98	1.47		
	4	−0.73	1.46		
	5	−0.51	1.83		
	6	−2.50	1.37		
	7	0.13	2.14		
	8	−2.03	1.89		
	9	−0.08	1.67		
	10	1.93	1.79		
P2_all_				−0.55	0.35

a Specification of groups is listed in Table 1.

b Baseline mean (BL) for baseline correction = mean of the last 30 movements in the PRE condition (out of 60 or else 120).

**Table A2.** Group Means and *SEM* Deviations in Degrees of the First Throw or Pointing Movement in the PRISM Condition (= Initial Prism Effect) and the First Throw or Pointing Movement in the POST Condition (= Initial Aftereffect) of the Throwing and Pointing Experiments. All Data are Baseline Corrected.

			Initial prism effect		Initial aftereffect	
Group	Prism^a^	*N*	Mean	*SEM*	Mean	*SEM*
T1	Right	8	12.0	1.1	−11.0	0.6
T2	Left	8	−11.4	0.6	10.5	1.0
T3	Right	10	13.8	0.5	−12.3	1.1
T4	Right	6	16.8	1.0	−9.0	1.5
T5	Left	6	−14.5	1.3	9.3	1.7
Tall	Right + Left	38	13.5	0.5	−10.7	0.5
P1	Right	10	14.4	1.1	−5.0	0.9
P2	Left	10	−6.6	2.0	10.5	1.0
Pall	Right + Left	20	10.5	1.4	−7.8	0.9

*Note. N* = number of subjects; *SEM* = standard error of the mean.

a Prism = Direction of prismatic shift.

**Table A3.** Averages and Grand Average of the Exponential Least Square Fits of All Groups. The Parameter *c* Corresponds to the Remaining Offset After an Infinite Number of Throws or Pointing Movements, Respectively as does the Parameter a in Table 2.

*f*(x) = *a* e*^bx^* + *c*			PRISM condition				POST condition			
Task	Group	Prism	*a* ^a^	*b*	***c*** **^a^**	Least square	*a* ^a^	*b*	***c*** **^a^**	Least square
Throwing	T1	R 17°	9.73	−0.07	**1.60**	0.90	−9.26	−0.10	−**0.21**	0.62
	T2	L 17°	−9.94	−0.11	−**0.53**	0.46	8.70	−0.08	**0.38**	0.40
	T3	R 17°	11.90	−0.03	**0.82**	0.51	−11.68	−0.07	−**0.61**	0.47
	T4	R 17°	9.96	−0.07	**0.96**	1.47	−8.73	−0.17	−**0.68**	0.77
	T5	L 17°	−13.34	−0.23	−**0.93**	1.23	6.54	−0.10	**0.28**	0.95
	T_all_		9.74	−0.07	**1.33**	0.37	−9.07	−0.09	−**0.50**	0.18
Pointing	P1	R 17°	21.13	−0.33	−**0.18**	0.37	−6.61	−0.17	−**0.44**	0.34
	P2	L 17°	−6.78	−0.10	−**0.37**	0.30	14.15	−0.42	**0.27**	0.34
	P_all_		12.96	−0.22	**0.15**	0.20	−8.87	−0.25	−**0.35**	0.16

a The values of *a* and *c* are in degrees (°).

**Table A4.** Group Means and *SEM* (=Standard Error of the Mean) of the Deviations of the Blocks (=30 Subsequent Movements) in the PRISM* and POST* Conditions of the *Throwing* and *Pointing* Experiments. Furthermore Are the According *t* and *p* Values Listed (One-Sided *t* Test; Uncorrected). All Data Are Baseline Corrected.

Group	Last block	*N*	Mean	*SEM*	*t*	*p* (one sided)	Significance
T1	Prism_B1	8	5.46	0.64	8.48	.0000	***
	Prism_B2	8	2.42	0.28	8.56	.0000	***
	Prism_B3	8	1.52	0.20	7.40	.0001	***
	Prism_B4	8	1.40	0.24	5.82	.0003	***
	Post_B1	8	−3.12	0.28	−11.30	.0000	***
	Post_B2	8	−0.77	0.14	−5.46	.0005	***
	Post_B3	8	−0.01	0.12	−0.07	.4727	Ø
	Post_B4	8	−0.01	0.14	−0.06	.4759	Ø
T2	Prism_B1	8	−3.40	0.29	−11.56	.0000	***
	Prism_B2	8	−0.85	0.30	−2.80	.0133	*
	Prism_B3	8	−0.34	0.27	−1.27	.1217	Ø
	Prism_B4	8	−0.47	0.27	−1.74	.0631	T
	Post_B1	8	3.46	0.38	9.13	.0000	***
	Post_B2	8	0.93	0.20	4.53	.0013	**
	Post_B3	8	0.31	0.16	1.93	.0474	*****
	Post_B4	8	0.25	0.12	2.02	.0416	*
T3	Prism_B1	10	8.17	0.83	9.78	.0000	***
	Prism_B2	10	4.06	1.17	3.46	.0036	**
	Prism_B3	10	2.23	0.89	2.50	.0168	*
	Prism_B4	10	1.38	0.51	2.70	.0122	*
	Prism_B5	10	0.90	0.31	2.91	.0087	**
	Prism_B6	10	0.85	0.32	2.63	.0137	*
	Prism_B7	10	0.86	0.27	3.19	.0055	**
	Prism_B8	10	0.67	0.29	2.35	.0215	*
	Post_B1	10	−5.13	0.40	−12.71	.0000	***
	Post_B2	10	−1.33	0.29	−4.53	.0007	**
	Post_B3	10	−0.45	0.13	−3.55	.0031	**
T4	Prism_B1	6	4.88	0.62	7.86	.0003	***
	Prism_B2	6	2.15	0.40	5.34	.0015	**
	Prism_B3	6	1.60	0.29	5.53	.0013	**
	Prism_B4	6	0.88	0.34	2.62	.0235	*
	Prism_B5	6	0.83	0.31	2.66	.0225	*
	Prism_B6	6	0.67	0.23	2.88	.0174	*
	Prism_B7	6	0.61	0.26	2.34	.0331	*
	Prism_B8	6	0.64	0.16	4.06	.0049	**
	Post_B1	6	−2.46	0.27	−9.22	.0001	***
	Post_B2	6	−0.78	0.28	−2.75	.0201	*
	Post_B3	6	−0.38	0.09	−4.12	.0046	**
T5	Prism_B1	6	−3.24	1.18	−2.75	.0202	*
	Prism_B2	6	−1.07	0.63	−1.71	.0742	T
	Prism_B3	6	−0.91	0.47	−1.95	.0542	T
	Prism_B4	6	−0.98	0.49	−2.01	.0501	T
	Prism_B5	6	−0.72	0.44	−1.62	.0828	T
	Prism_B6	6	−0.84	0.44	−1.89	.0587	T
	Prism_B7	6	−0.74	0.33	−2.24	.0374	*
	Prism_B8	6	−0.63	0.40	−1.56	.0902	*
	Post_B1	6	2.27	1.09	2.08	.0459	*
	Post_B2	6	0.20	0.62	0.32	.3798	Ø
	Post_B3	6	0.35	0.30	1.14	.1532	Ø
T_all_	Prism_B1	38	5.30	0.45	11.83	.0000	***
	Prism_B2	38	2.27	0.38	5.90	.0000	***
	Prism_B3	38	1.30	0.28	4.64	.0000	***
	Prism_B4	38	1.05	0.18	5.79	.0000	***
	Post_B1	38	−3.48	0.28	−12.56	.0000	***
	Post_B2	38	−0.86	0.15	−5.90	.0000	***
	Post_B3	38	−0.30	0.07	−4.05	.0001	***
P1	Prism_B1	10	1.83	0.62	2.96	.0080	**
	Prism_B2	10	−0.40	0.52	−0.76	.2341	Ø
	Prism_B3	10	−0.14	0.48	−0.29	.3888	Ø
	Prism_B4	10	−0.19	0.46	−0.42	.3432	Ø
	Post_B1	10	−1.57	0.50	−3.12	.0062	**
	Post_B2	10	−0.31	0.42	−0.73	.2410	Ø
	Post_B3	10	−0.44	0.38	−1.15	.1389	Ø
	Post_B4	10	−0.63	0.44	−1.43	.0933	T
P2	Prism_B1	10	−2.42	0.80	−3.02	.0072	**
	Prism_B2	10	−0.66	0.16	−4.13	.0013	**
	Prism_B3	10	−0.37	0.27	−1.39	.0988	T
	Prism_B4	10	−0.18	0.29	−0.61	.2791	Ø
	Post_B1	10	1.42	0.38	3.74	.0023	**
	Post_B2	10	0.36	0.23	1.54	.0789	T
	Post_B3	10	0.11	0.18	0.58	.2873	Ø
	Post_B4	10	0.11	0.20	0.55	.2975	Ø
P_all_	Prism_B1	20	2.13	0.50	4.28	.0002	***
	Prism_B2	20	0.13	0.29	0.45	.3290	Ø
	Prism_B3	20	0.12	0.27	0.42	.3383	Ø
	Prism_B4	20	−0.01	0.27	−0.03	.4881	Ø
	Post_B1	20	−1.49	0.31	−4.86	.0001	***
	Post_B2	20	−0.33	0.23	−1.43	.0847	*
	Post_B3	20	−0.27	0.21	−1.31	.1035	Ø
	Post_B4	20	−0.37	0.24	−1.52	.0721	T

*Note.* Prism = adaptation period with prisms on; Post = readaptation period without prisms; T = trend < .1.

Blocks are 30 subsequent movements (B1 = movement 1–30; B2 = movement 31–60, etc.).

* *p* < .05. ***p* < .01. ****p* < .001.
