# Supplementary material for: Limited Plasticity of Prismatic Visuomotor Adaptation
Source: Iperception. 2017 Apr 10;8(2):2041669517701458. doi: 10.1177/2041669517701458 (PMC5407531; doi:10.1177/2041669517701458)
Supplement: Supplementary material [file IPE701458_supplementary_material.pdf]

Supplementary material

Table S1. Individual means (+STD) and Group means (+SEM) of the deviations of all observers in the PRE condition used for baseline correction of the *Throwing* and *Pointing* Experiments.

Pre Condition<br>Group [a]	Subject	Baseline mean[b]	Baseline STD	Group BL mean	Group BL SEM
**T1**	1	-0.60	1.95		
	2	0.24	2.23		
	3	-0.40	1.95		
	4	-0.14	1.90		
	5	-0.19	2.12		
	6	-1.24	2.48		
	7	-0.12	1.89		
	8	-0.10	1.40		
**T1 all**				**-0.32**	**0.17**
**T2**	1	0.00	2.12		
	2	-0.25	1.30		
	3	-0.26	1.02		
	4	0.12	2.67		
	5	-0.32	1.57		
	6	-1.20	1.36		
	7	-0.38	1.20		
	8	0.53	1.72		
**T2 all**				**-0.22**	**0.19**
**T3**	1	-0.61	1.71		
	2	0.16	1.87		
	3	-0.14	1.75		
	4	0.23	1.69		
	5	-0.03	4.90		
	6	0.17	1.55		
	7	0.07	1.54		
	8	0.02	1.19		
	9	-0.34	1.64		
	10	-0.28	1.34		
**T3 all**				**-0.08**	**0.09**

**T4**	1	-0.22	2.33		
	2	0.75	2.42		
	3	-0.65	3.34		
	4	0.10	2.17		
	5	-0.68	1.71		
	6	-0.38	2.15		
**T4 all**				**-0.18**	**0.24**
**T5**	1	-0.62	2.75		
	2	-0.24	2.62		
	3	0.86	2.25		
	4	0.01	2.26		
	5	-0.43	2.38		
	6	0.39	3.61		
**T5 all**				**-0.01**	**0.25**
**P1**	1	2.63	2.07		
	2	-2.00	1.14		
	2	0.75	1.49		
	4	0.34	1.72		
	5	-3.56	1.83		
	6	-2.58	1.76		
	7	1.14	1.21		
	8	-2.79	1.83		
	9	-2.86	1.52		
	10	-2.17	1.31		
**P1 all**				**-1.11**	**0.71**
**P2**	1	-0.65	1.85		
	2	-0.22	1.64		
	2	0.98	1.47		
	4	-0.73	1.46		
	5	-0.51	1.83		
	6	-2.50	1.37		
	7	0.13	2.14		
	8	-2.03	1.89		
	9	-0.08	1.67		
	10	1.93	1.79		
**P2 all**				**-0.55**	**0.35**

[a] Specification of groups is listed in Table 1.
[b] Baseline mean (BL) for baseline correction = mean of the last 30 movements in the PRE condition (out of 60 or else 120).

Table S2. Group means and SEM deviations in degrees of the first throw or pointing movement in the PRISM condition (= initial prism effect) and the first throw or pointing movement in the POST condition (= initial aftereffect) of the Throwing and Pointing Experiments. All data are baseline-corrected.

Group	Prism[a]	N[b]	Initial prism effect		Initial aftereffect	
			Mean	SEM[c]	Mean	SEM
T1	R	8	12.0	1.1	-11.0	0.6
T2	L	8	-11.4	0.6	10.5	1.0
T3	R	10	13.8	0.5	-12.3	1.1
T4	R	6	16.8	1.0	-9.0	1.5
T5	L	6	-14.5	1.3	9.3	1.7
Tall	R+L	38	13.5	0.5	-10.7	0.5
P1	R	10	14.4	1.1	-5.0	0.9
P2	L	10	-6.6	2.0	10.5	1.0
Pall	R+L	20	10.5	1.4	-7.8	0.9

[a] Prism= Direction of prismatic shift
[b] N=number of subjects
[c]SEM= standard error of the mean

Table S3. Averages and grand average of the exponential least square fits of all groups. The parameter "c" corresponds to the remaining offset after an infinite number of throws or pointing movements respectively.

$f(x) = a e^{bx} + c$			PRISM condition				POST condition			
**Task**	**Group**	**Prism**	a[a]	b	**c[a]**	least squa.	a[a]	b	**c[a]**	least squa.
**Throwing**	T1	R 17°	9.73	-0.07	**1.60**	0.90	-9.26	-0.10	**-0.21**	0.62
	T2	L 17°	-9.94	-0.11	**-0.53**	0.46	8.70	-0.08	**0.38**	0.40
	T3	R 17°	11.90	-0.03	**0.82**	0.51	-11.68	-0.07	**-0.61**	0.47
	T4	R 17°	9.96	-0.07	**0.96**	1.47	-8.73	-0.17	**-0.68**	0.77
	T5	L 17°	-13.34	-0.23	**-0.93**	1.23	6.54	-0.10	**0.28**	0.95

	T all		9.74	-0.07	**1.33**	0.37	-9.07	-0.09	**-0.50**	0.18
**Pointing**	P1	R 17°	21.13	-0.33	**-0.18**	0.37	-6.61	-0.17	**-0.44**	0.34
	P2	L 17°	-6.78	-0.10	**-0.37**	0.30	14.15	-0.42	**0.27**	0.34
	P all		12.96	-0.22	**0.15**	0.20	-8.87	-0.25	**-0.35**	0.16

[a] a,c are in °.

Table S4. Group means and SEM (=standard error of the mean) of the deviations of the Blocks (=30 subsequent movements) in the PRISM* and POST* conditions of the *Throwing* and *Pointing* Experiments. Furthermore are the according t and p-values listed (one-sided t-Test; uncorrected). All data are baseline-corrected.

Group	last Block	N	Mean	SEM	t	p (one-sided)	significance
T1	Prism_B1	8	5.46	0.64	8.48	0.0000	***
	Prism_B2	8	2.42	0.28	8.56	0.0000	***
	Prism_B3	8	1.52	0.20	7.40	0.0001	***
	Prism_B4	8	1.40	0.24	5.82	0.0003	***
	Post_B1	8	-3.12	0.28	-11.30	0.0000	***
	Post_B2	8	-0.77	0.14	-5.46	0.0005	***
	Post_B3	8	-0.01	0.12	-0.07	0.4727	Ø
	Post_B4	8	-0.01	0.14	-0.06	0.4759	Ø
T2	Prism_B1	8	-3.40	0.29	-11.56	0.0000	***
	Prism_B2	8	-0.85	0.30	-2.80	0.0133	*
	Prism_B3	8	-0.34	0.27	-1.27	0.1217	Ø
	Prism_B4	8	-0.47	0.27	-1.74	0.0631	T
	Post_B1	8	3.46	0.38	9.13	0.0000	***
	Post_B2	8	0.93	0.20	4.53	0.0013	**
	Post_B3	8	0.31	0.16	1.93	0.0474	*
	Post_B4	8	0.25	0.12	2.02	0.0416	*
T3	Prism_B1	10	8.17	0.83	9.78	0.0000	***
	Prism_B2	10	4.06	1.17	3.46	0.0036	**
	Prism_B3	10	2.23	0.89	2.50	0.0168	*
	Prism_B4	10	1.38	0.51	2.70	0.0122	*
	Prism_B5	10	0.90	0.31	2.91	0.0087	**
	Prism_B6	10	0.85	0.32	2.63	0.0137	*
	Prism_B7	10	0.86	0.27	3.19	0.0055	**

	Prism_B8	10	0.67	0.29	2.35	0.0215	*
	Post_B1	10	-5.13	0.40	-12.71	0.0000	***
	Post_B2	10	-1.33	0.29	-4.53	0.0007	**
	Post_B3	10	-0.45	0.13	-3.55	0.0031	**
T4	Prism_B1	6	4.88	0.62	7.86	0.0003	***
	Prism_B2	6	2.15	0.40	5.34	0.0015	**
	Prism_B3	6	1.60	0.29	5.53	0.0013	**
	Prism_B4	6	0.88	0.34	2.62	0.0235	*
	Prism_B5	6	0.83	0.31	2.66	0.0225	*
	Prism_B6	6	0.67	0.23	2.88	0.0174	*
	Prism_B7	6	0.61	0.26	2.34	0.0331	*
	Prism_B8	6	0.64	0.16	4.06	0.0049	**
	Post_B1	6	-2.46	0.27	-9.22	0.0001	***
	Post_B2	6	-0.78	0.28	-2.75	0.0201	*
	Post_B3	6	-0.38	0.09	-4.12	0.0046	**
T5	Prism_B1	6	-3.24	1.18	-2.75	0.0202	*
	Prism_B2	6	-1.07	0.63	-1.71	0.0742	T
	Prism_B3	6	-0.91	0.47	-1.95	0.0542	T
	Prism_B4	6	-0.98	0.49	-2.01	0.0501	T
	Prism_B5	6	-0.72	0.44	-1.62	0.0828	T
	Prism_B6	6	-0.84	0.44	-1.89	0.0587	T
	Prism_B7	6	-0.74	0.33	-2.24	0.0374	*
	Prism_B8	6	-0.63	0.40	-1.56	0.0902	*
	Post_B1	6	2.27	1.09	2.08	0.0459	*
	Post_B2	6	0.20	0.62	0.32	0.3798	Ø
	Post_B3	6	0.35	0.30	1.14	0.1532	Ø
Tall	Prism_B1	38	5.30	0.45	11.83	0.0000	***
	Prism_B2	38	2.27	0.38	5.90	0.0000	***
	Prism_B3	38	1.30	0.28	4.64	0.0000	***
	Prism_B4	38	1.05	0.18	5.79	0.0000	***
	Post_B1	38	-3.48	0.28	-12.56	0.0000	***
	Post_B2	38	-0.86	0.15	-5.90	0.0000	***
	Post_B3	38	-0.30	0.07	-4.05	0.0001	***
P1	Prism_B1	10	1.83	0.62	2.96	0.0080	**

	Prism_B2	10	-0.40	0.52	-0.76	0.2341	Ø
	Prism_B3	10	-0.14	0.48	-0.29	0.3888	Ø
	Prism_B4	10	-0.19	0.46	-0.42	0.3432	Ø
	Post_B1	10	-1.57	0.50	-3.12	0.0062	**
	Post_B2	10	-0.31	0.42	-0.73	0.2410	Ø
	Post_B3	10	-0.44	0.38	-1.15	0.1389	Ø
	Post_B4	10	-0.63	0.44	-1.43	0.0933	T
P2	Prism_B1	10	-2.42	0.80	-3.02	0.0072	**
	Prism_B2	10	-0.66	0.16	-4.13	0.0013	**
	Prism_B3	10	-0.37	0.27	-1.39	0.0988	T
	Prism_B4	10	-0.18	0.29	-0.61	0.2791	Ø
	Post_B1	10	1.42	0.38	3.74	0.0023	**
	Post_B2	10	0.36	0.23	1.54	0.0789	T
	Post_B3	10	0.11	0.18	0.58	0.2873	Ø
	Post_B4	10	0.11	0.20	0.55	0.2975	Ø
Pall	Prism_B1	20	2.13	0.50	4.28	0.0002	***
	Prism_B2	20	0.13	0.29	0.45	0.3290	Ø
	Prism_B3	20	0.12	0.27	0.42	0.3383	Ø
	Prism_B4	20	-0.01	0.27	-0.03	0.4881	Ø
	Post_B1	20	-1.49	0.31	-4.86	0.0001	***
	Post_B2	20	-0.33	0.23	-1.43	0.0847	*
	Post_B3	20	-0.27	0.21	-1.31	0.1035	Ø
	Post_B4	20	-0.37	0.24	-1.52	0.0721	T

Prism= adaptation period with prisms on; Post= readaptation period without prisms.

Blocks are 30 subsequent movements (B1= movement 1-30; B2=movement 31-60 etc.).

Significances are marked with *** for p< .001. ** for p< .01. * for p< .05. T=trend < .1.
